# Resource Allocation and Interference Coordination Strategies in Heterogeneous Dual-Layer Satellite Networks

**DOI:** 10.3390/s25041005

**Published:** 2025-02-08

**Authors:** Jinhong Li, Rong Chai, Tianyi Zhou, Chengchao Liang

**Affiliations:** 1 School of Communication & Information Engineering, Chongqing University Posts & Telecommunications, Chongqing 400065, China; d180101003@stu.cqupt.edu.cn (J.L.); zhoutianyicqupt@163.com (T.Z.); 2 Key Laboratory of Intelligent Computing for Advanced Manufacturing, Chongqing University Posts & Telecommunications, Chongqing 400065, China

**Keywords:** heterogeneous satellite networks, interference coordination, LEO satellite networks, resource allocation

## Abstract

In the face of rapidly evolving communication technologies and increasing user demands, traditional terrestrial networks are challenged by the need for high-quality, high-speed, and reliable communication. This paper explores the integration of heterogeneous satellite networks (HSN) with emerging technologies such as Mobile Edge Computing (MEC), in-network caching, and Software-Defined Networking (SDN) to enhance service efficiency. By leveraging dual-layer satellite networks combining Low Earth Orbit (LEO) and Geostationary Earth Orbit (GEO) satellites, the study addresses resource allocation and interference coordination challenges. This paper proposes a novel resource allocation and interference coordination strategy for dual-layer satellite networks integrating LEO and GEO satellites. We formulate a mathematical optimization problem to optimize resource allocation while minimizing co-channel interference and develop an ADMM-based distributed algorithm for efficient problem-solving. The proposed scheme enhances service efficiency by incorporating MEC, in-network caching, and SDN technologies into the satellite network. Simulation results demonstrate that our proposed algorithm significantly improves network performance by effectively managing resources and reducing interference.

## 1. Introduction

Research into sixth-generation (6G) networks has gained significant traction, particularly as the traffic continues its rapid evolution, reaching 5016 EB by 2030 toward more complex and demanding applications [1]. Meanwhile, ongoing work on resource allocation in heterogeneous 5G networks emphasizes scalability, adaptive user provisioning, and emerging deployment challenges [2], providing valuable insights into how future 6G systems can extend or refine these strategies. In parallel, aerospace integrated networks—often envisioned as a critical component for 6G—promise global connectivity through interconnected satellite constellations and airborne platforms [3]. LEO satellites not only provide computing and content retrieval services but are also capable of efficient data transmission, particularly in scenarios sensitive to time delays. In contrast, GEO satellites primarily function as data transmission relays, offering stable connectivity over wider areas. Building on these advances, our study presents a novel dual-layer satellite architecture designed to synchronize communication and computation resources across both LEO and GEO satellites, with the ultimate goal of empowering next-generation IoT services.

With the rapid advancement of science and technology, human civilization is continually progressing. Today, human activities are no longer limited to plains; they have expanded into deserts, oceans, the sky, and even outer space. However, these areas are characterized by wide distribution and high density, which makes information transmission in these areas somewhat challenging. Due to the broad spatial distribution, long transmission distances, and limited node capacity, traditional cellular systems can no longer meet the demand for real-time communication. Satellite communication, with its large communication capacity, long transmission distance, and ability to ignore complex topographical features, has gained increasing attention in recent years and offers new solutions to these issues [4]. Specifically, dual-layer satellite networks can further enhance the stability and reliability of satellite communication, as well as expand bandwidth and coverage. By integrating multi-layer satellite and ground networks, the geographical limitations of ground networks can be overcome, making heterogeneous satellite networks (HSN) a popular development direction in modern communication technology [5].

However, deploying HSN introduces critical challenges that require innovative solutions. Key issues include efficient resource allocation to meet diverse user demands, minimizing co-channel interference between LEO and GEO satellites in shared spectrum scenarios, and ensuring low-latency service delivery for applications such as video streaming. These challenges are further compounded by the complexity of integrating emerging technologies like Mobile Edge Computing (MEC), in-network caching, and Software-Defined Networking (SDN) into HSN architectures.

According to the [6], the development of the industry has enriched short video content, significantly increasing user numbers and making it a primary source of mobile internet usage time and traffic. As of December 2023, the user size of online video in China had reached 1.067 billion. The ubiquity of video services presents a huge challenge for the design and operation of the next generation of mobile networks. One of the issues faced by video transmission services is the environmental limitations and resource scarcity of terrestrial cellular networks, which can cause disruptions and instability in video service transmission. Considering the characteristics of heterogeneous satellite networks discussed earlier, introducing them into video service transmission schemes presents new possibilities.

There are still several challenges in implementing video transmission services in heterogeneous satellite networks. First, although there have been numerous innovations in the wireless communication and networking fields over the past few years, allowing mobile users to access cloud data at extremely fast transmission rates, the current network architecture still suffers from significant propagation delays, which are unacceptable for latency-sensitive applications. For example, in video applications, retrieving video resources from the cloud can lead to long initial buffering times and video playback stuttering. Second, while the emergence of heterogeneous satellite networks has expanded the network’s reach, it has also increased its complexity. Therefore, when transmitting video streams in heterogeneous satellite networks, it is necessary to measure and predict user traffic and proactively allocate and plan network resources based on the network status to ensure users’ video quality demands and reduce the pressure on network operations and maintenance. Finally, the simultaneous presence of multiple types of networks will lead to spectrum resource scarcity, severely limiting the future development of heterogeneous satellite networks. Given the non-renewable nature of spectrum resources, spectrum sharing is one of the inevitable solutions [7]. However, spectrum sharing also introduces new issues, such as co-channel interference between heterogeneous networks, especially the interference of low Earth orbit satellite networks with high Earth orbit satellite networks, because high orbit satellites have absolute priority to ensure their service quality is not affected by low orbit satellites [8,9]. Therefore, in heterogeneous satellite networks based on video stream transmission, resource allocation and interference coordination still require further research.

Compared to traditional terrestrial cellular networks, heterogeneous satellite networks that integrate MEC, in-network caching, and SDN can provide more efficient services to users. In this network, users can first request services from edge nodes equipped with caching and computing capabilities. If the edge nodes cannot meet the user’s needs, the user can still access cloud server resources through the satellite backhaul network. The entire network’s traffic planning and resource scheduling are uniformly controlled by SDN technology. However, due to the complexity of heterogeneous networks and the mix of resources, how to allocate resources to meet each user’s requests still requires further research. Additionally, the issue of co-channel interference in heterogeneous satellite networks also needs to be addressed. Thus, the paper proposes a heterogeneous satellite network integrating MEC, in-network caching, and SDN to enhance service efficiency over traditional networks. The main contributions are summarized as follows.

- This paper proposes to tackle resource allocation and interference coordination challenges in dual-layer satellite networks combining LEO and GEO satellites.
- A mathematical optimization problem is formulated to optimize resource allocation while minimizing co-channel interference. An ADMM-based distributed algorithm is proposed to solve the optimization problem efficiently.
- The algorithm decomposes the problem into subproblems for users, service nodes, and the network, allowing distributed optimization. The incorporation of MEC (Mobile Edge Computing), in-network caching, and SDN (Software-Defined Networking) enhances service efficiency in the satellite network.
- Simulation results show that the proposed algorithm improves network performance by efficiently managing resources and reducing interference.

The paper is structured as follows. Section 2 reviews existing literature on heterogeneous networks, satellite communications, and resource allocation strategies, emphasizing the need for integrated network architectures. Section 3 presents the formulation of the resource allocation and interference coordination problem within heterogeneous dual-layer satellite networks. Section 4 introduces the algorithm designed to solve the formulated problem, detailing its methodology and theoretical underpinnings. Section 5 describes the simulation setup and parameters used and analyzes the performance outcomes of the proposed algorithm. Section 6 summarizes the key findings and discusses potential future research directions.

## 2. Related Works

This section outlines the current research related to resource allocation and interference coordination strategies in heterogeneous networks, summarized in Table 1.

### 2.1. Resource Allocation Strategies in Heterogeneous Networks

With the widespread application of video services in heterogeneous networks, addressing the allocation of video resources has become particularly important. Using MEC and in-network caching technologies, data can be cached at network nodes to reduce network load and enhance user experience. Meanwhile, SDN technology can help coordinate complex network conditions while minimizing the impact on transmission capacity. The application of these technologies can improve the performance and stability of video services while also saving costs and reducing bandwidth demands. It is anticipated that with continuous technological advancements, the application of video services in heterogeneous satellite networks will become more widespread, bringing more innovation and development.

In [10], Li et al. focus on enhancing energy efficiency and traffic offloading in integrated satellite/terrestrial radio access networks, showing that carefully designed resource allocation strategies can significantly improve overall network performance. This insight is pertinent to our work, as we also target efficient resource allocation—albeit with an emphasis on mitigating interference in dual-layer satellite systems. Meanwhile, ref. [11] considers a joint communication, computing, and caching (3C) paradigm in LEO satellite MEC networks. Their 3C approach aligns with our proposal of leveraging edge computing and in-network caching, illustrating how distributed caching can reduce latencies—a critical aspect when supporting time-sensitive video traffic. Regarding SDN-based frameworks, ref. [12] demonstrates how software-defined platforms enable flexible and adaptive video streaming over HTTP. Our work builds on these SDN concepts to dynamically orchestrate resources between LEO and GEO satellites, ensuring that network adjustments can be made swiftly as user demands shift.

In [10], Li et al. focus on enhancing [13] highlights femtocaching, in which distributed caching helpers are deployed to offload data and reduce backbone congestion. This idea of distributed content storage complements our use of in-network caching within heterogeneous satellite networks, aiming to alleviate bottlenecks and ensure smoother video delivery to end users. The literature [14] considers the limited caching capacity of base stations and proposes an optimization problem in the RAN considering both backhaul and wireless resources, achieving a video-aware caching strategy to maximize user QoE and network utility. Additionally, with the rapid development of internet social media platforms, video services like short video sharing have seen explosive growth. To speed up video sharing and extend transmission distances, video caching technology has been expanded to satellites. Based on this, the literature [15] proposes a coverage-aware collaborative video caching algorithm that considers the popularity of videos within the coverage area and the cooperation between neighboring satellites.

While a lot of work has been done on MEC-enabled integrated satellite networks, most of it views the satellite network as a relay network. Directly processing tasks on satellites without losing general applicability can further enhance user QoE. The study in [16] thoroughly explores the issue of utilizing MEC technology to improve the resource utilization and service assurance capabilities of integrated satellite networks. The work in [17] combines storage and computing resources in MEC servers to support users in selecting video streams of different bitrate levels. It then designs base station caching strategies and user association schemes based on various storage capacities, computing power, and downlink bandwidth resources.

To tackle changing network conditions and diverse user preferences, adaptive bitrate streaming has become a key technology in mobile video streaming. The paper [18] introduces the concept of MEC applications aimed at enhancing users’ overall experience quality when accessing streaming services through popular video caching management and video quality adaptation. The paper [19] proposes a method combining the advantages of in-network caching and MEC to boost mobile network throughput and user QoE. This method also puts forward a flexible transcoding strategy to provide users with low-latency video streaming services in MEC networks, given limited storage, computing, and spectrum resources.

In the study [20], the authors focus on the flow table management issue in software-defined satellite-terrestrial networks and propose a multi-strategy flow table management scheme. The paper [21] also introduces a software-defined satellite-terrestrial integrated network for the joint management and orchestration of network, caching, and computing resources. The authors of [22] propose a traffic engineering scheme in software-defined RAN based on real-time video traffic, addressing the downlink transmission multipath traffic engineering problem under backhaul and wireless access constraints to achieve greater throughput and QoE gains. The paper [22] first introduces an online method for dynamically estimating the effective rate of video streams to provide a satisfactory quality of experience. Second, it proposes a traffic engineering method that considers the characteristics of video streams. Finally, the paper discusses a radio coordination method for providing stable video rates across cells. The study [23] presents a novel mechanism that jointly considers buffer dynamics, video quality adaptation, in-network caching, video transcoding, and transmission to optimize energy consumption and QoE metrics in video streaming.

In summary, existing research has demonstrated that implementing edge computing technology, in-network caching, and software-defined networking in heterogeneous satellite networks can further enhance network performance. Additionally, these technologies have shown effectiveness in video services to some extent. However, current studies lack research on the integration of heterogeneous satellite networks, edge caching, in-network caching, and software-defined networking for video services, which requires further in-depth exploration.

### 2.2. Interference Coordination Strategies in Heterogeneous Networks

Currently, most satellites launched into space are LEO satellites, followed by GEO satellites. As the number of satellites grows rapidly, the integration of LEO and GEO satellite systems is becoming an inevitable trend. However, spectrum sharing between LEO and GEO can lead to co-channel interference, causing communication anomalies. To assess the level of interference from Non-Geostationary Orbit (NGEO) satellite constellations on GEO constellations, Equivalent Power Flux Density (EPFD) is widely used. Many studies use EPFD as the interference threshold for LEO satellites on GEO satellites, and when this threshold is exceeded, appropriate remedial measures need to be taken. The studies [24,25] examine the scenarios of GEO–LEO coexistence systems in both uplink and downlink interference environments. The research indicates that when both satellite systems use the same frequency band for communication, co-channel interference occurs in both uplink and downlink.

To tackle interference issues between GEO and LEO satellites, the International Telecommunication Union Radiocommunication Sector (ITU-R S)1419 introduces several interference suppression techniques for co-channel LEO and GEO systems, such as power control, the use of high-gain antennas, setting up protection zones for ground stations, satellite resource scheduling, link balancing, and geographic isolation [26]. ITU-R S.1430 explains how NGEO satellite ground stations can set up protection zones [27]. ITU-R S.1655 discusses interference suppression schemes for frequency sharing between GEO and NGEO systems in the 37.5–42.5 GHz and 47.2–50.2 GHz bands, including satellite resource scheduling and setting up isolation zones [9].

In satellite communications, to prevent interference from LEO satellites to GEO satellite ground users, an “Exclusion Zone” (EZ) is often set on LEO satellites, forcing them to shut off their beams in those areas. The study [28] proposes a static spectrum-sharing method that achieves spectrum sharing between LEO and GEO satellites by limiting communication between LEO ground stations and LEO satellites. In the LEO-GEO coexistence scenario in [25], the authors implement an exclusive angle strategy, setting isolation angles to reduce co-linear downlink interference from LEO satellites to GEO satellite users. The study based on EPFD analysis [29,30,31] determines the range of angular separation LEO satellites need to adopt to take avoidance measures. Although these schemes can ensure that the GEO satellite systems are kept within interference limits, they impose certain constraints on the communication of LEO satellite ground mobile terminals. Ensuring service quality of GEO satellite networks by controlling the beam switching of all LEO satellites is challenging in practical applications.

Power control is a commonly used method to suppress interference. It manages the interference intensity received by controlling the power output of the interfering transmitter. In the study [32], a beam power control scheme for GEO and LEO satellite coexistence systems is proposed. This scheme aims to maximize the throughput of LEO satellite users under the constraint of GEO satellite users’ service quality. However, before executing the algorithm, the scheme does not plan the frequency of LEO satellites, which may lead to overlaps of multiple co-channel beams, thus reducing the communication capacity of LEO satellites. The study [33] proposes a method for spectrum sharing between LEO and GEO satellites achieved through cooperation among LEO satellites. This method utilizes multi-layer LEO constellations, allowing a user to be served by up to four LEO satellites simultaneously. However, it introduces co-channel beam interference among different LEO satellites, increasing the complexity of interference analysis and leading to resource waste, therefore driving up costs. The study [34] presents a novel heterogeneous framework to combine GEO and LEO systems to enhance uplink spectral efficiency. This framework uses two satellites simultaneously to receive signals from ground users and employs non-orthogonal multiple access techniques for frequency resource multiplexing. The reference [35] proposes a dynamic spectrum-sharing method, which continuously optimizes power allocation to ensure that the services of low Earth orbit satellites do not affect the service quality of GEO beams.

Although the ITU and some scholars have studied the interference between LEO and GEO satellites, most research is based on isolated interference scenarios without incorporating specific business contexts, highlighting the need for further study.

## 3. System Model and Problem Formulation

### 3.1. Network Model

**Figure 1:**
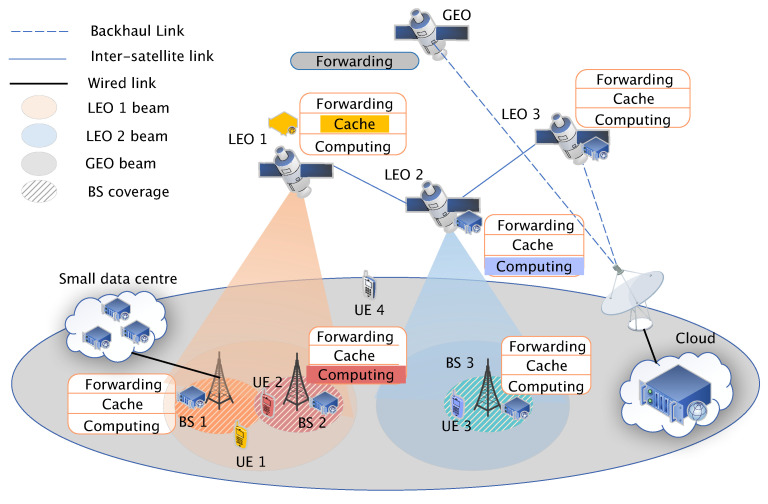
Heterogeneous Satellite Network Scene Model.

As illustrated in Figure 1, the network comprises an access network and a backhaul network. The access network consists of base stations and Multi-access Edge Computing (MEC) servers. MEC servers, with computational capabilities and caching space, can be deployed at base stations or form small data centers. To simplify, MEC servers at the same location are abstracted as a single edge server representing a small data center. Base stations are connected to nearby small data centers via wired links. The backhaul network consists of one GEO satellite and multiple LEO satellites. LEO satellites host servers that provide computation and content retrieval services, while the GEO satellite acts solely as a data transmission relay. Satellites communicate with ground stations equipped with satellite transceivers via wireless links. In Figure 1, UE 1 receives caching services from LEO 1, while UE 2 is served by BS 2, which provides computing services. Given that BS 3 may be unable to supply the necessary computing services, UE 3 opts for LEO 2 for computing needs. As UE 4 falls outside the coverage of both BS and LEO, it resorts to using GEO to transmit its data to the ground cloud.

This network can be modeled as a directed graph $G\left(D,L\right)$, where D represents the set of nodes and L represents the set of links. The node set D includes users M and service nodes N (base stations, small data centers, satellites, and cloud servers). For network nodes without deployed servers, the caching and computational capacities are set to zero. To distinguish access nodes from service nodes, an access point set J⊂N is defined, comprising all base stations *j*. The set of links L includes all wireless and wired links.

To support user video services, nodes must cooperate and share resources to ensure smooth video data stream transmission, which may traverse multiple paths. This paper’s scheme aims to determine the set of paths for all video streams by solving the proposed algorithm. Initially, a candidate path set for users is constructed. Let $P_{m}$ denote user *m*’s candidate paths, and $P_{mn}\subset P_{m}$ represent candidate paths from node *n* to user *m*. The complete set of candidate paths for all users forms the path set P.

Constructing the candidate path set relies primarily on the adjacency matrix for candidate service and relay nodes. The relay nodes’ adjacency matrix is established during network setup. Selecting candidate service nodes involves matching user demands with the cached content at service nodes. Specifically, a hit event $h_{mn}=1$ indicates that user *m* has been successfully matched with service node *n*, therefore designating node *n* as a candidate service node for user *m*. Path $p_{mn}^{k}\in P_{m}$ represents the *k*-th path from service node *n* to user *m*. If this path is selected, the data rate of this path is $r_{mn}^{k}\in {ℜ}^{+}$. Therefore, the achievable rate of user *m* is the aggregate rate of all selected paths, i.e., $\sum_{p_{mn}^{k}\in P_{m}}r_{mn}^{k}$.

According to the *Article 5.523A* of the ITU Radio Regulations [29], Non-Geostationary Orbit (NGSO) satellites can share the 18.8–19.3 GHz and 28.6–29.1 GHz frequency bands with geostationary orbit (GEO) satellites, provided they do not cause unacceptable interference to GEO satellites. To ensure compliance, the ITU has established an EPFD limit [29].

**Figure 2:**
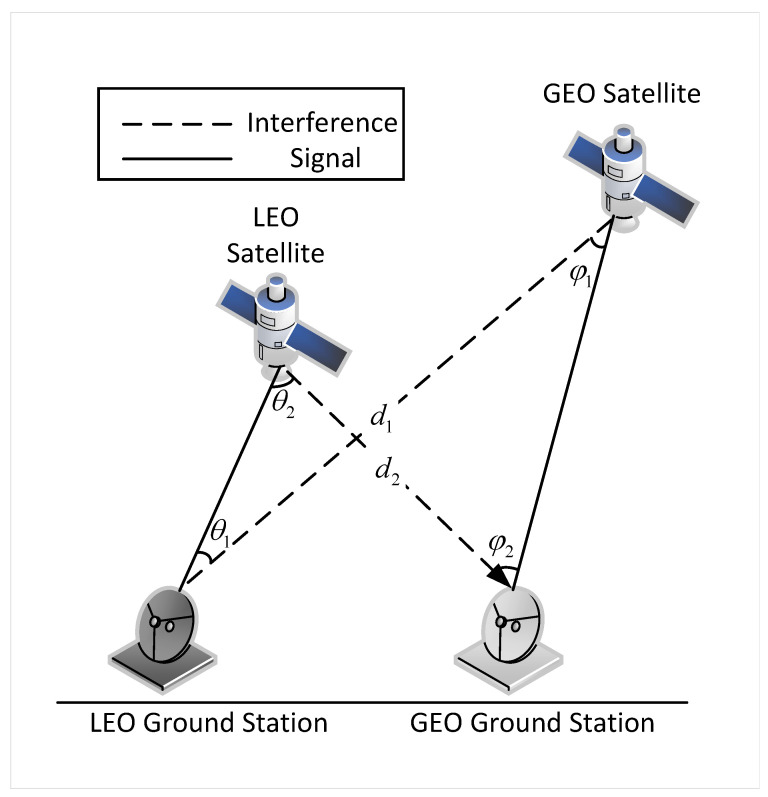
Schematic diagram of antenna off-axis angle and interference distance.

EPFD is the aggregate power flux density received by a ground station or a GEO orbit receiving station from all transmitting stations within the NGSO satellite system, accounting for the off-axis discrimination of a reference receiving antenna. The ITU specifies the EPFD value, which is controlled by LEO satellites to limit interference to GEO satellites; it is not determined through GEO–LEO satellite interaction. The EPFD expression is given as follows [30,31]:

(1) $$\begin{array}{c} EPFD=10\log\left(\sum_{i=1}^{N}p_{i}\frac{G_{t}\left(\theta_{i}\right)}{4\pi d_{i}^{2}}\cdot \frac{G_{r}\left(\varphi_{i}\right)}{G_{r\max}}\right) \end{array}$$

where $p_{i}$ represents the transmit power of the LEO satellite, $G_{t}\left(\theta_{i}\right)$ is the antenna gain of the LEO satellite at off-axis angle $\theta_{i}$, $G_{r}\left(\varphi_{i}\right)$ is the antenna gain of the GEO satellite ground station at off-axis angle $\varphi_{i}$, $d_{i}$ is the distance between the LEO satellite and the GEO satellite ground station, and $G_{r\max}$ is the maximum gain of the receiving antenna. Figure 2 illustrates the off-axis angles θ and φ, and the interference distance *d*. As shown in Equation (1), the interference EPFD received by the GEO satellite is inversely proportional to the interference distance and off-axis angles and directly proportional to the transmit power. Since the distance and off-axis angles can be calculated based on the physical positions of the nodes, the EPFD value can be adjusted by controlling the transmit power.

Table 2 lists the EPFD thresholds set by the ITU, where the carrier bandwidth for interference scenarios is 40 kHz.

As the paper focuses on video retrieval, this paper considers only the downlink EPFD. The scenario particularly involves interference signals from a LEO satellite captured at a GEO ground station. For each GEO ground station *j*, the EPFD value must adhere to the following constraint. These ground stations, which are equipped for both transmitting to and receiving from GEO satellites, are denoted by $j\in J_{geo}$.

(2) $$\begin{array}{c} EPFD_{j}\leq EPFD_{th},\;\forall j\in J_{geo} \end{array}$$

User terminals connect to network nodes via wireless links, enabling communication between users and the network, with all communication routed through access points. In this paper, the wireless access points are referred to as edge base stations *j*, where users access the resource network by connecting through these base stations to acquire the desired video files. For the wireless network system, define the binary control variable $x_{mj}\in \left\{0,1\right\}$, indicating the connection status between user *m* and base station *j*. If $x_{mj}=1$, user *m* has chosen base station *j* as their wireless access point; if not, then $x_{mj}=0$. In contemporary mobile networks, a user typically connects to a single wireless access point. Thus, the following constraint enforces the user’s selection of an access base station:

(3) $$\begin{array}{c} \sum_{j\in J}x_{mj}=1,\;\forall m\in M \end{array}$$

Each user is limited to a single connection to an access point; therefore, any video stream data intended for user *m* must initially be delivered to the access base station *j* linked to user *m*. From there, it is relayed by base station *j* directly to user *m*. Consequently, the wireless link between the user and the access base station is tasked with conveying the user’s entire video data stream. This situation gives rise to specific traffic limitations for the wireless links:

(4) $$\begin{array}{c} \sum_{p_{mn}^{k}\in P_{m}}a_{mj}r_{mn}^{k}=x_{mj}\sum_{g=1}^{G}z_{gm}v_{g},\;\forall m\in M,j\in J \end{array}$$

where $a_{mj}$ signifies whether the data stream utilizes the path from base station *j* to user *m*. If this path is utilized, then $a_{mj}=1$; otherwise, $a_{mj}=0$. This connectivity constraint secures the full route for the video data stream from the service node to the user, ensuring that user *m*’s video data stream is sent to the access base station before being delivered to the user itself.

### 3.2. Joint Problem Formulations

In accordance with the node service model, let us define the computational resource allocation variable $y_{mn}\in {ℜ}^{+}$, which signifies the computational resources assigned by service node *n* to user *m*. This paper presumes a computational model that entails simultaneous data processing and transmission; the service node transmits pre-processed data while continuing computations. Therefore, in this paper, $y_{mn}$ denotes the speed at which transcoding services operate, i.e., the rate of output of computational results per second. If $y_{mn}<r_{mn}$, part of the available data link capacity remains unused; conversely, if $y_{mn}>r_{mn}$, user *m* may not receive data from service node *n* promptly, degrading the user’s quality of experience. To enhance the efficiency of logical link utilization and improve the user’s vMOS, the following constraint should be adhered to:

(5) $$\sum_{p_{mn}^{k}\in P_{mn}}r_{mn}^{k}=h_{mn}y_{mn},\;\forall m\in M,n\in N$$

To fulfill the computational demands for varying video resolution quality levels required by distinct users, it is essential for the network to allocate the correct amount of computational resources. Moreover, to optimize the utilization of these resources at the service nodes, the computational resources across all service nodes must align with the transcoding needs specific to each user. The computational resources necessary for transcoding are directly linked to the data rate for the chosen resolution level by the user. To ensure this alignment, the following condition must be satisfied:

(6) $$\sum_{g=1}^{G}z_{gm}c_{g}=\sum_{n\in N}y_{mn},\;\forall m\in M,n\in N$$

Besides the limits on link power capacity discussed earlier, network design must also take into account the following physical resource constraints: restrictions on wired connections, wireless connections, and the computational capacity at service nodes. These factors have a significant impact on both network performance and reliability. Specifically, the following conditions must be met:

(7) $$\sum_{p_{mn}^{k}\in P_{l}}a_{l}r_{mn}^{k}\leq B_{l},\;\forall l\in L_{wd}$$

(8) $$\sum_{p_{mn}^{k}\in P_{l}}a_{l}r_{mn}^{k}\leq {\hat{R}}_{l}^{\max},\;\forall l\in L_{wl}$$

(9) $$\sum_{m\in M}y_{mn}\leq C_{n},\;\forall n\in N$$

The goal of this study is to enhance the network’s overall efficiency, with the average vMOS for users chosen as the utility function. Taking into account the previously outlined constraints, the problem, denoted as P0, is formulated as follows:

(10) $$\begin{array}{cccc} P0:\; & \min_{Z,X,Y,R,\hat{P}} & \; & -\frac{1}{\left|M\right|}\sum_{m\in M}\sum_{g=1}^{G}s_{g}z_{gm}\\ \; & s.\;t.\; & C1:\; & \sum_{g=1}^{G}z_{gm}=1,\;\forall m\in M\\ \; & \; & C2:\; & \sum_{j\in J}x_{mj}=1,\;\forall m\in M\\ \; & \; & C3:\; & \sum_{p_{mn}^{k}\in P_{mn}}r_{mn}^{k}=h_{mn}y_{mn},\;\forall m\in M,n\in N\\ \; & \; & C4:\; & \sum_{g=1}^{G}z_{gm}c_{g}=\sum_{n\in N}y_{mn},\;\forall m\in M\\ \; & \; & C5:\; & \sum_{p_{mn}^{k}\in P_{m}}a_{mj}r_{mn}^{k}=x_{mj}\sum_{g=1}^{G}z_{gm}v_{g},\;\forall m\in M,j\in J\\ \; & \; & C6:\; & EPFD_{j}\leq EPFD_{th},\;\forall j\in J_{geo}\\ \; & \; & C7:\; & \sum_{m\in M}y_{mn}\leq C_{n},\;\forall n\in N\\ \; & \; & C8:\; & \sum_{l\in L_{n}}2^{{\hat{P}}_{l}}\leq P_{n},\;\forall n\in N\\ \; & \; & C9:\; & \sum_{p_{mn}^{k}\in P_{l}}a_{l}r_{mn}^{k}\leq B_{l},\;\forall l\in L_{wd}\\ \; & \; & C10:\; & \sum_{p_{mn}^{k}\in P_{l}}a_{l}r_{mn}^{k}\leq {\hat{R}}_{l}^{\max},\;\forall l\in L_{wl}\\ \; & \; & C11:\; & z_{gm},x_{mj}\in \left\{0,1\right\},r_{mn}^{k},p_{l},y_{mn}\in {ℜ}^{+} \end{array}$$

## 4. ADMM-Based Distributed Solution Strategy

In optimization problem P0, constraint C11 indicates that the variables $z_{gm}$ and $x_{mj}$ must be binary, making the problem a mixed-integer nonlinear programming challenge. Moreover, there is a coupling of variables in constraints C3, C4, C5, and C10, adding complexity to finding a solution. To enhance efficiency, a more detailed analysis and refinement of the problem are necessary.

### 4.1. Problem Transformation

To elucidate the product relationship between the variables $z_{gm}$ and $x_{mj}$ in constraint C5, this section introduces a novel variable $f_{gm}^{j}=z_{gm}x_{mj}$. This variable denotes whether user *m* selects a video with resolution level *g* and connects via access point *j*. Consequently, constraint C5 is reformulated as follows:

(11) $$\sum_{p_{mn}^{k}\in P_{m}}a_{mj}r_{mn}^{k}=\sum_{g=1}^{G}f_{gm}^{j}v_{g},\;\forall m\in M,j\in J$$

Upon the introduction of a new variable $f_{gm}^{j}$, the numerical constraint of C2 remains unaltered; however, the focus shifts from users selecting a single access base station to each user picking one access base station specifically for video streams at resolution level *g*. The constraint concerning variable $x_{mj}$ within C2 is thus reformulated as follows:

(12) $$\sum_{j\in J}\sum_{g=1}^{G}x_{mj}z_{gm}=1,\;\forall m\in M$$

This adjustment, centered around the newly defined variable $f_{gm}^{j}=z_{gm}x_{mj}$, modifies constraint C2 further into:

(13) $$\sum_{j\in J}\sum_{g=1}^{G}f_{gm}^{j}=1,\;\forall m\in M$$

Both are subsequently relaxed to take values in the interval [0,1]. Consequently, constraint C8 is updated to:

(14) $$z_{gm},f_{gm}^{j}\in \left[0,1\right],r_{mn}^{k},p_{l},y_{mn}\in {ℜ}^{+}$$

With these transformations, the original optimization problem P0 is converted into a new problem, denoted as $P0^{\prime }$, expressed as:

(15) $$\begin{array}{cccc} P0^{\prime }:\; & \min_{Z,F,Y,R,\hat{P}} & \; & -\frac{1}{\left|M\right|}\sum_{m\in M}\sum_{g=1}^{G}s_{g}z_{gm}\\ \; & s.\;t.\; & C1:\; & \sum_{g=1}^{G}z_{gm}=1,\;\forall m\in M\\ \; & \; & C2:\; & \sum_{j\in J}\sum_{g=1}^{G}f_{gm}^{j}=1,\;\forall m\in M\\ \; & \; & C3:\; & \sum_{p_{mn}^{k}\in P_{mn}}r_{mn}^{k}=h_{mn}y_{mn},\;\forall m\in M,n\in N\\ \; & \; & C4:\; & \sum_{g=1}^{G}z_{gm}c_{g}=\sum_{n\in N}y_{mn},\;\forall m\in M\\ \; & \; & C5:\; & \sum_{p_{mn}^{k}\in P_{m}}a_{mj}r_{mn}^{k}=\sum_{g=1}^{G}f_{gm}^{j}v_{g},\;\forall m\in M,j\in J\\ \; & \; & C6:\; & EPFD_{j}\leq EPFD_{th},\;\forall j\in J_{geo}\\ \; & \; & C7:\; & \sum_{m\in M}y_{mn}\leq C_{n},\;\forall n\in N\\ \; & \; & C8:\; & \sum_{l\in L_{n}}2^{{\hat{P}}_{l}}\leq P_{n},\;\forall n\in N\\ \; & \; & C9:\; & \sum_{p_{mn}^{k}\in P_{l}}a_{l}r_{mn}^{k}\leq B_{l},\;\forall l\in L_{wd}\\ \; & \; & C10:\; & \sum_{p_{mn}^{k}\in P_{l}}a_{l}r_{mn}^{k}\leq {\hat{R}}_{l}^{\max},\;\forall l\in L_{wl}\\ \; & \; & C11:\; & z_{gm},f_{gm}^{j}\in \left[0,1\right],r_{mn}^{k},{\hat{p}}_{l},y_{mn}\in {ℜ}^{+} \end{array}$$

### 4.2. Problem Decomposition and Joint Optimization Algorithm

In this paper, the Alternating Direction Method of Multipliers (ADMM) [36] is utilized to address the interdependencies between variables in $P0^{\prime }$. This approach breaks down the problem into subproblems focused on the user side, the service node side, and the network side, solving each independently to enhance efficiency. In detail, the user side is responsible for selecting video resolution levels and choosing access points; the service node side oversees the allocation of computational resources; and the network side manages the planning of transmission paths and the allocation of power for wireless links.

To construct the augmented Lagrangian function for the problem $P0^{\prime }$, we first establish independent local feasible sets $\Pi_{z,f}$, $\Pi_{y}$, and $\Pi_{r,\hat{p}}$ for the variables in each sub-problem as given below:

(16) $$\left\{\begin{array}{cc} \; & \Pi_{z,f}=\left\{\left\{z_{gm},f_{gm}^{j}\right\}|\left[0,1\right],\mathrm{constraints}\;C1,C2\right\}\\ \; & \Pi_{y}=\left\{\left\{y_{mn}\right\}|R^{+},\mathrm{constraint}\;C7\right\}\\ \; & \Pi_{r,\hat{p}}=\left\{\left\{r_{mn}^{k},{\hat{p}}_{l}\right\}|R^{+},\mathrm{constraints}\;C6,C8,C9,C10\right\} \end{array}\right.$$

To address the coupling constraints, we introduce dual variables $\lambda_{mn}$, $\nu_{m}$, and $\mu_{mj}$ for the relaxation of constraints C3, C4, and C5, respectively. This yields the following augmented Lagrangian function:

(17) $$\begin{array}{cc} L_{\rho }\left(Z,F,Y,R,\hat{P},\lambda ,\nu ,\mu \right) & =-U\left(Z\right)+\sum_{m\in M}\sum_{n\in N}\lambda_{mn}L_{\rho_{1}}+\sum_{m\in M}\nu_{m}L_{\rho_{2}}\\ \; & +\sum_{m\in M}\sum_{j\in J}\mu_{mj}L_{\rho_{3}}+\frac{\rho_{1}}{2}\sum_{m\in M}\sum_{n\in N}L_{\rho_{1}}^{2}\\ \; & +\frac{\rho_{2}}{2}\sum_{m\in M}L_{\rho_{2}}^{2}+\frac{\rho_{3}}{2}\sum_{m\in M}\sum_{j\in J}L_{\rho_{3}}^{2} \end{array}$$

where $\rho_{1}$, $\rho_{2}$, and $\rho_{3}\geq 0$ act as penalty parameters, and $L_{\rho_{1}}$, $L_{\rho_{2}}$, and $L_{\rho_{3}}$ are specified by:

(18) $$\left\{\begin{array}{cc} \; & L_{\rho_{1}}=\sum_{p_{mn}^{k}\in P_{mn}}r_{mn}^{k}-h_{mn}y_{mn}\\ \; & L_{\rho_{2}}=\sum_{g=1}^{G}z_{gm}c_{g}-\sum_{n\in N}y_{mn}\\ \; & L_{\rho_{3}}=\sum_{p_{mn}^{k}\in P_{m}}a_{mj}r_{mn}^{k}-\sum_{g=1}^{G}f_{gm}^{j}v_{g} \end{array}\right.$$

**Figure 3:**
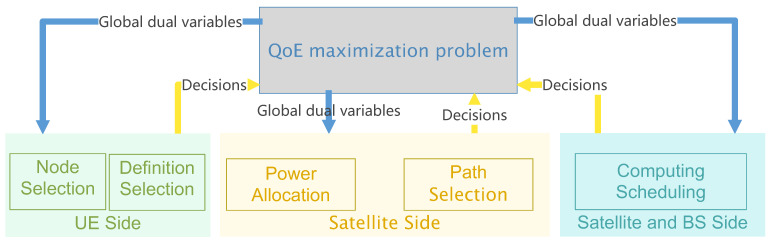
Problem Decomposition.

By utilizing ADMM, we can switch between optimizing the objective function and separating the problem $P0^{\prime }$ into three subproblems: the video resolution and access node selection issue on the user side, the computational scheduling challenge on the service node side, and the path planning and power allocation on the network side. In particular, when tackling the user-side issue, the variables related to the service node side and network side are kept fixed, and the same approach is applied to the other subproblems. After completing the optimization, the centralized controller treats the outcomes of the distributed optimization as fixed values to address the overall problem from the controller’s perspective. The algorithm ceases iterations once the stopping conditions are satisfied. This scheme is summarized in Figure 3.

The issue of video resolution and selection of an access node can be handled separately by each user on their own. Consequently, the individual user’s optimization problem is formulated as follows:

(19) $$\begin{array}{cc} P1: & \min_{z_{gm},f_{gm}^{j}\in \left[0,1\right]}-\frac{1}{\left|M\right|}\sum_{g=1}^{G}s_{g}z_{gm}+\nu_{m}L_{\rho_{2}}+\sum_{j\in J}\mu_{mj}L_{\rho_{3}}\\ \; & \;+\frac{\rho_{2}}{2}L_{\rho_{2}}^{2}+\frac{\rho_{3}}{2}\sum_{j\in J}L_{\rho_{3}}^{2}\\ s.t. & \;C1:\;\sum_{g=1}^{G}z_{gm}=1\\ \; & \;C2:\;\sum_{j\in J}\sum_{g=1}^{G}f_{gm}^{j}=1 \end{array}$$

In problem P1, the goal involves a standard convex objective function along with two linear equality constraints. Due to these characteristics, the problem can be effectively tackled using convex optimization tools such as CVX [37].

Analogous to the computational scheduling issue on the user side, the service node-side scheduling challenge can likewise be addressed independently by each service node. The problem specific to each service node is expressed as:

(20) $$\begin{array}{cc} P2: & \min_{y_{mn}\in {ℜ}^{+}}\sum_{m\in M}\sum_{n\in N}\lambda_{mn}L_{\rho_{1}}+\sum_{m\in M}\nu_{m}L_{\rho_{2}}\\ \; & \;+\frac{\rho_{1}}{2}\sum_{m\in M}\sum_{n\in N}L_{\rho_{1}}^{2}+\frac{\rho_{2}}{2}\sum_{m\in M}L_{\rho_{2}}^{2}\\ s.t. & \;C7:\;\sum_{m\in M}y_{mn}\leq C_{n},\;\forall n\in N \end{array}$$

The issue of path planning and power allocation on the network side is structured as follows:

(21) $$\begin{array}{cc} P3: & \min_{r_{mn}^{k},{\hat{p}}_{l}\in {ℜ}^{+}}\sum_{m\in M}\sum_{n\in N}\lambda_{mn}L_{\rho_{1}}+\sum_{m\in M}\sum_{j\in J}\mu_{mj}L_{\rho_{3}}\\ \; & +\frac{\rho_{1}}{2}\sum_{m\in M}\sum_{n\in N}L_{\rho_{1}}^{2}+\frac{\rho_{3}}{2}\sum_{m\in M}\sum_{j\in J}L_{\rho_{3}}^{2}\\ s.t. & C6:\;EPFD_{j}\leq EPFD_{th},\;\forall j\in J_{geo}\\ \; & C8:\;\sum_{l\in L_{n}}2^{{\hat{p}}_{l}}\leq P_{n},\;\forall n\in N\\ \; & C9:\;\sum_{p_{mn}^{k}\in P_{l}}\alpha_{l}r_{mn}^{k}\leq B_{l},\;\forall l\in L_{wd}\\ \; & C10:\;\sum_{p_{mn}^{k}\in P_{l}}\alpha_{l}r_{mn}^{k}\leq {\hat{R}}_{l}^{\max},\;\forall l\in L_{wl} \end{array}$$

In every cycle *t*, after addressing the distributed subproblems, the dual variables are revised according to the following expressions:

(22) $$\left\{\begin{array}{cc} \; & \lambda_{mn}\left(t+1\right)=\lambda_{mn}\left(t\right)-\rho_{1}\left(\sum_{p_{mn}^{k}\in P_{mn}}r_{mn}^{k}-h_{mn}y_{mn}\right)\\ \; & \nu_{m}\left(t+1\right)=\nu_{m}\left(t\right)-\rho_{2}\left(\sum_{g=1}^{G}z_{gm}c_{gm}-\sum_{n\in N}y_{mn}\right)\\ \; & \mu_{mj}\left(t+1\right)=\mu_{mj}\left(t\right)-\rho_{3}\left(\sum_{p_{mn}^{k}\in P_{m}}a_{mj}r_{mn}^{k}-\sum_{g=1}^{G}f_{gm}^{j}v_{g}\right) \end{array}\right.$$

After breaking down the problem, we can view it as having two parts: the user side and the network side. On the user side, video resolution levels are selected based on the resources provided by the network. On the network side, resources are allocated based on the user’s choices. If the video resolution level selected by the user remains unchanged, the network side’s demand will not change either. Once the resource demand on the network side cannot be met, the network will notify the video user to lower the resolution demand through network pricing. Therefore, if this paper can solve the internal problems P1, P2, and P3 in each iteration, the SDN controller can update the dual variables and pass them to the nodes and users, helping the nodes and users find the optimal solution to their subproblems. These subproblems can each be optimized independently, considering their unique local constraints and dual variables. Specifically:

- User-side optimization: Users select the suitable video resolution and access point from the available network resources.
- Service node-side optimization: Service nodes distribute computational resources to users.
- Network-side optimization: The network decides on transmission paths and administers power for the wireless links.

Once these subproblems are solved, the dual variables are updated, and the cycle is repeated until the system converges. The subsequent algorithm outlines the ADMM-based distributed approach for managing resource distribution and coordinating interference within the GEO–LEO satellite system.

After solving the problem, the user’s video resolution level $z_{gm}$ and the new variable $f_{gm}^{j}$ are converted back to binary variables based on the marginal benefits of the obtained linear solution. Here, considering that the new variable $f_{gm}^{j}$ represents the selection of access base station *j* for the video file requested by user *m* at level *g*, it is necessary to first determine the video quality level *g* chosen by user *m* based on $z_{gm}$ before obtaining the access node selection variable $x_{mj}$. Then, find the submatrix corresponding to $\left\{f_{gm}^{j}\right\}$ based on parameters *m* and *g*, which is the set corresponding to the access node selection variable $x_{mj}$. The complete algorithm flow is shown in Algorithm 1.
**Algorithm 1** Algorithm for ADMM-based Resource Allocation and Interference Coordination1:**Input:** Network (D,L), user video demand *f*.2:**Initialization** Initialize variables Z(0), X(0), Y(0), R(0), and $\hat{P}\left(0\right)$, as well as dual variables $\lambda_{mn}\left(0\right)$, $\nu_{m}\left(0\right)$, and $\mu_{mj}\left(0\right)$. Set stopping threshold ϵ and maximum iterations *T*.3:**for** each step t≤T **do**4:   Broadcast dual variables $\lambda_{mn}\left(t\right)$, $\nu_{m}\left(t\right)$, and $\mu_{mj}\left(t\right)$ from the SDN controller to users and service nodes.5:   Solve the user-side optimization problem P1 for each user to obtain updated video resolution Z(t+1) and access point selection F(t+1).6:   Solve the service node-side optimization problem P2 to obtain updated computational resource allocation Y(t+1).7:   Solve the network-side optimization problem P3 to update transmission paths R(t+1) and power allocation $\hat{P}\left(t+1\right)$.8:   Update the dual variables $\lambda_{mn}\left(t+1\right)$, $\nu_{m}\left(t+1\right)$, and $\mu_{mj}\left(t+1\right)$ using the results from step 4.9:   **if** the difference in the objective function value between iterations *t* and t−1 is less than ϵ **then**10:     **stop**11:   **end if**12:**end for**13:**Output:** Video resolution selection Z, access point selection X, resource allocation Y, transmission paths R, and power allocation $\hat{P}$.

To make the proposed scheme more accessible, consider a simplified dual-layer satellite scenario with two users (U1 and U2), one LEO satellite acting as a service node with limited computational and caching resources, one GEO satellite functioning purely as a communication relay, and one ground base station connected to a small data center. We assume:

- Each user requests video service at one of two resolution levels (e.g., HD or SD).
- The LEO satellite and data center can process and cache video content, while the GEO satellite primarily forwards traffic.
- Spectrum resources must be shared among all links, and interference arises if both satellites transmit in the same frequency band simultaneously.

We can formulate a smaller version of our optimization model for this scenario as follows:

- Decision variables: xU1,xU2∈0,1 to indicate whether each user is assigned to SLEO or BS for primary video access. rU1,rU2 to represent the chosen resolution levels (e.g., 1 for SD, 2 for HD).
- Objective: Maximize overall video quality while minimizing interference and respecting power/bandwidth constraints at SLEO and BS.
- Constraints: Each user must be served by either SLEO or BS (but not both). The LEO satellite has limited power and needs to avoid interfering with GEO satellite transmissions. When the user’s resolution (rUi) remains fixed, the network side must ensure sufficient resource allocation for that demand. If resources become constrained, the network (SDN controller) may signal one or both users to downgrade their resolutions to ensure stable service.

Although modest in scale, this example highlights how our scheme coordinates resource allocation among multi-layer satellite links and ground stations. By solving the user-side and network-side subproblems with ADMM, we iteratively assign service nodes, video resolution levels, and transmission parameters in a way that balances performance, interference mitigation, and resource limitations. Scaling up to more satellites, users, and base stations follows the same core logic while adding corresponding subproblems and constraints.

### 4.3. Algorithm Performance Analysis

The problem $P0^{\prime }$ is a convex optimization problem featuring both a convex objective function and constraints, thus ensuring convergence to a globally optimal solution. Moreover, the augmented Lagrangian approach supports strong duality, meaning that the ADMM-based algorithm achieves convergence to the global optimum.

Regarding complexity analysis, utilizing centralized methods like primal-dual interior-point algorithms to solve $P0^{\prime }$ would entail a complexity of $O\left({\left(MNKL\right)}^{3}\right)T_{1}$, where $T_{1}$ denotes the number of iterations, *M* is the user count, *N* the number of service nodes, *K* the number of candidate paths, and *L* represents the number of links. However, by applying ADMM for problem decomposition, complexity is notably diminished, allowing for distributed solving at the level of each component (user, service node, and network).

The complexity for resolving the user-side sub-problem P1 via a convex optimization tool is $O\left({\left(MGJ\right)}^{3}\right)$, where *G* stands for the number of video quality levels, and *J* the number of access points. The service node-side sub-problem P2 has a complexity of $O\left({\left(MN\right)}^{3}\right)$. Lastly, solving the network-side sub-problem P3 carries a complexity of $O\left({\left(MNKL\right)}^{3}\right)T_{1}$.

Consequently, the overall complexity of each iteration of the proposed ADMM-based algorithm is $O\left({\left(MNKL\right)}^{3}\right)T_{1}$. With $T_{2}$ iterations for achieving convergence, the total complexity becomes $O\left({\left(MNKL\right)}^{3}\right)T_{1}T_{2}$. This highlights that the ADMM algorithm can substantially reduce computational complexity while ensuring effective resource allocation and interference coordination in a GEO–LEO satellite network.

In addition to providing a detailed complexity analysis of our ADMM-based algorithm, we compared its computational efficiency with two commonly referenced methods in the literature.

Centralized Interior-Point Method. Classic interior-point solvers applied to similar optimization problems demonstrate a polynomial time complexity that grows significantly with the problem size $O\left({\left(MNKL\right)}^{3}\right)$ per iteration. While these solvers can converge in relatively few iterations, they rely on a centralized structure, leading to high memory overhead and potentially long solution times for large-scale heterogeneous satellite networks.

Gradient-Based Distributed Approach. Distributed gradient descent or primal-dual methods operate without forming large Hessian matrices, reducing per-iteration costs. However, these methods often need more iterations to achieve convergence and may suffer from slow progress in the presence of highly coupled constraints, as found in dual-layer satellite networks.

By decomposing the resource allocation and interference coordination problem into subproblems (user side, service node side, and network side), the ADMM-based algorithm strikes a balance between per-iteration complexity and convergence speed. Consequently, the total complexity per iteration is lower than that of the centralized interior-point approach, while convergence is typically faster than standard gradient-based methods in scenarios with tightly coupled constraints. This efficiency is particularly advantageous for large-scale GEO–LEO satellite networks.

## 5. Simulation Results and Analysis

In this section, MATLAB is used to simulate and analyze the performance of the proposed ADMM-based resource allocation and interference coordination strategy. The simulated network consists of multiple edge nodes and backhaul networks, with edge networks composed of multiple users, access points, and small data centers. Users communicate with access points via wireless links, while access points and small data centers are connected via wired links. The backhaul network comprises multiple LEO and GEO satellites, where access points can request resources from the cloud via the satellite network or communicate with other access points.

The satellite-earth propagation was modeled to accurately reflect real-world communication conditions. The following factors were considered. Free-space path loss (FSPL) is calculated as follows.

(23) $$FSPL\left(dB\right)=20\log_{10}\left(d\right)+20\log_{10}\left(f\right)+32.45$$

where *d* is the distance between the satellite and the ground station in kilometers, and *f* is the frequency in MHz. Atmospheric attenuation and rain attenuation are modeled based on using the ITU-R P.618-13 recommendation to account for signal degradation [38].

The network service nodes are pre-loaded with video resources from the cloud, cached within the network. Each user randomly requests a video file, and the following parameters, based on ITU’s EPFD limits, are used for the simulation, as shown in Table 3. The bandwidth values for LEO and GEO satellites were chosen based on the frequency bands typically allocated for satellite communications, such as the Ku-band (12–18 GHz) and Ka-band (26.5–40 GHz), as per ITU-R recommendations. These bands are widely used in satellite networks for high-speed data transmission. The transmission power levels were selected to balance the trade-off between maintaining adequate signal strength and minimizing interference. The values align with power levels reported in recent studies on LEO-GEO coexistence.

In this paper, the specific values chosen for the satellite parameters (Table 3) reflect realistic operational thresholds and commonly referenced industry standards. For instance, the LEO orbit height of 1200 km represents a practical trade-off between reduced latency and wide coverage, aligning with typical commercial LEO constellations; the GEO orbit height of 35,786 km corresponds to the standard geostationary ring, ensuring minimal relative orbital motion from the ground perspective. Similarly, the inclination angle of 87° approximates near-polar orbits often employed to achieve global coverage. Antenna gains for both LEO and GEO satellites (25 dBi) and ground stations (45 dBi for LEO, 30 dBi for GEO) are derived from typical engineering designs in the literature, balancing transmit power demands with hardware constraints. These selections ensure that our simulation environment is both realistic and representative of state-of-the-art satellite network deployments, thus strengthening the applicability of our resource allocation and interference coordination findings.

In this paper, we compare our proposed scheme with a baseline and other peers [11,39,40].

- Proposed: the scheme proposed in this paper.
- Cache only: the scheme proposed in [39] where only cache is deployed.
- Satellite only: the scheme proposed in [11] where only the satellites have caching and computing capability.
- Ground only: the scheme proposed in [40] where only the satellites have caching and computing capability.
- Baseline: the scheme that the network nodes only provide forwarding service.

**Figure 4:**
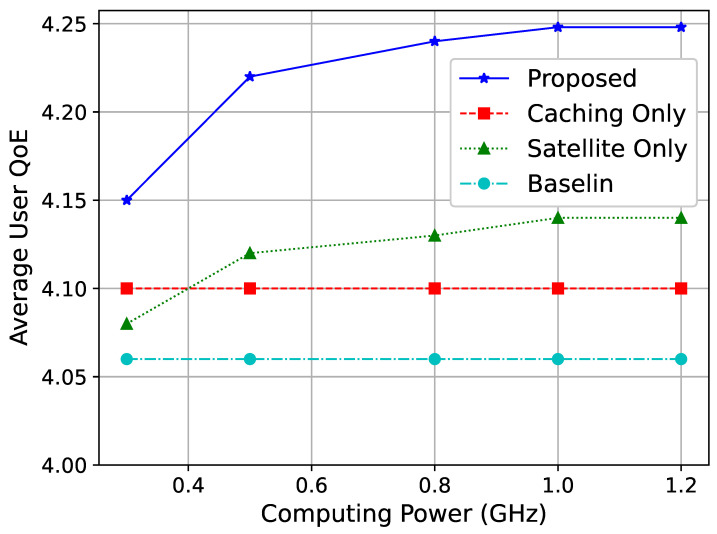
Average user vMOS for different computing capacities.

Figure 4 shows the average user vMOS under different normalized computing capacities. The normalized computing capacity refers to the ratio of the tested computing resource capacity to the default value. As seen in the figure, as the computing capacity increases, the overall performance of the network improves. This is because higher computing power enables the network nodes to handle more tasks, allowing more users to retrieve video resources nearby, thus improving network performance. Moreover, the observed QoE improvement is attributed to the efficient allocation of resources and effective interference coordination. By optimizing power control and frequency allocation, our algorithm minimizes co-channel interference between GEO and LEO satellites. This leads to enhanced spectral efficiency and higher data rates for users. Specifically, the dynamic adjustment of resource allocation in response to network conditions ensures that users experience consistent and improved service quality, even as the network scales.

When the computing capacity reaches a certain threshold, the proposed scheme begins to stabilize. This is due to other limiting factors in the network, such as cache hit rates and link resources, preventing further performance improvement. The stabilization of performance at higher computing capacities (as seen in Figure 4) indicates diminishing returns beyond a certain threshold. This insight can guide network planners in optimizing resource allocation by balancing computing capacity with other factors, such as caching and link bandwidth, to achieve cost-effective deployments.

**Figure 5:**
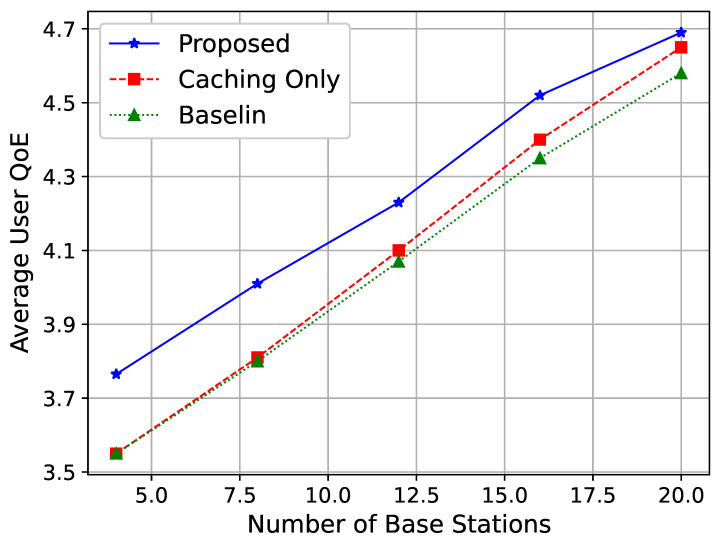
Average user vMOS for different numbers of access points.

Figure 5 shows the relationship between the number of access points (APs) and the average vMOS of users. It can be observed that as the number of access points increases, the average vMOS of users improves. This is because an increase in the number of access points enhances the network’s service capacity by providing more resources and offering better access environments for users. Additionally, more access points improve network coverage and channel conditions, further enhancing user experience. Increasing the number of base stations reduces the number of users served by each base station. This alleviates traffic congestion, allowing each user to experience better service quality due to more available resources per user. More base stations enable the network to implement frequency reuse more effectively. By assigning the same frequency bands to non-adjacent cells, the network maximizes spectral efficiency while minimizing co-channel interference. This efficient use of spectrum resources contributes to higher data throughput and improved QoE.

**Figure 6:**
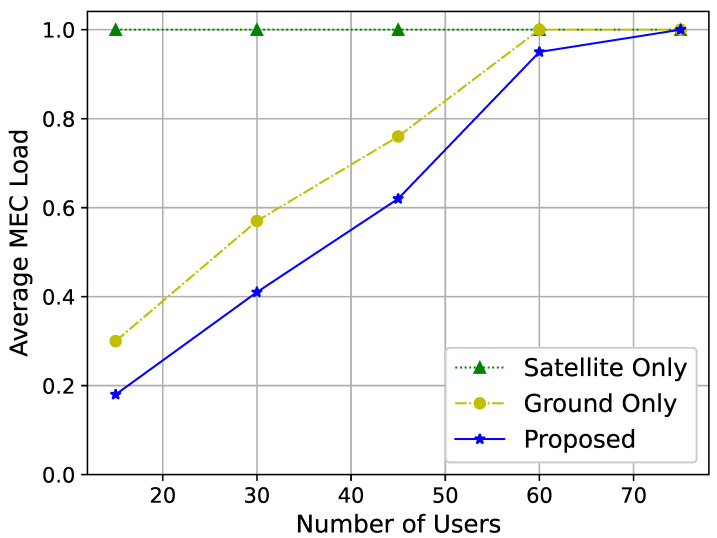
Average MEC server load for different numbers of users.

**Figure 7:**
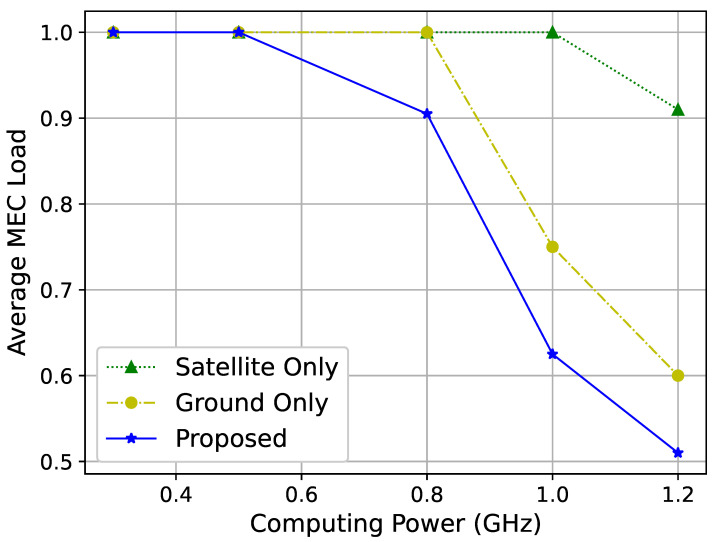
Average MEC server load for different cache capacities.

**Figure 8:**
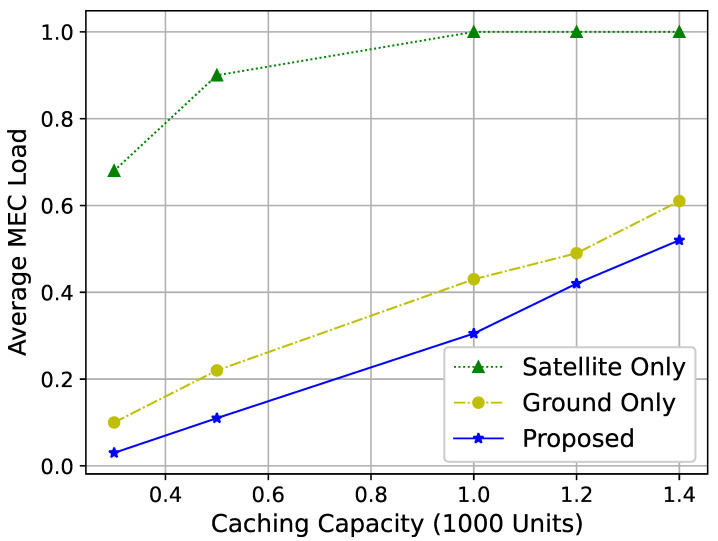
Average MEC server load for different computing capacities.

Next, Figure 6, Figure 7 and Figure 8 illustrate the average MEC server load under different network settings. To examine the impact of network architecture, we compare a solution where MEC servers are only deployed at terrestrial base stations and another where MEC servers are only deployed at satellites.

Figure 6 shows the relationship between the number of users and the average load on MEC servers. In the satellite service solution, the average load on MEC servers is consistently high due to the extensive coverage area of satellites. As the number of users increases, the MEC server load also increases due to the additional tasks the network needs to handle. Thus, as the number of users increases, the MEC server load continues to rise.

Moreover, Figure 5 and Figure 6 demonstrate the effectiveness of the proposed algorithm in minimizing co-channel interference between GEO and LEO satellites. This is particularly critical in heterogeneous satellite networks where spectrum sharing is inevitable. The results suggest that the proposed strategy can enable more efficient spectrum utilization while maintaining service quality. In practice, this means that satellite operators can adopt the proposed algorithm to support higher user densities and data rates without compromising network stability.

Figure 7 demonstrates the relationship between the cache capacity of service nodes and the average load on MEC servers. As cache capacity increases, the MEC server load also increases. This is because service nodes with higher cache capacities store more video files, improving the hit rate and increasing the likelihood that users retrieve video resources from service nodes, therefore increasing the computation load on the MEC servers.

Figure 8 shows the relationship between the computational capacity of MEC servers and their average load. As the computing power increases, the load on MEC servers gradually decreases. This is because although more tasks can be handled with greater computational power, the number of users and their demand remains constant in this simulation. Hence, once the computational capacity surpasses the demand, the average load on MEC servers decreases.

The proposed interference coordination strategy is integral to the ADMM-based algorithm, as it mitigates co-channel interference among multiple satellites and user terminals. By coordinating resource usage, the algorithm ensures that interference levels remain within acceptable limits, therefore enhancing overall network performance. Simulation results indicate that without effective interference coordination, the network experiences increased latency and reduced throughput, underscoring the necessity of this strategy in achieving optimal resource allocation.

The improvements in user QoE, as shown in Figure 7 and Figure 8, underscore the practical benefits of dynamic resource allocation and interference coordination. For instance, the ability to dynamically adjust resource allocation based on network conditions ensures consistent service quality, even under varying traffic loads. This is particularly relevant for applications such as live video streaming and remote sensing, where maintaining high QoE is critical for user satisfaction and operational success. The inherent advantages of LEO satellites, including their ability to perform data transmission with lower latency, make them particularly advantageous for applications such as live video streaming and real-time data processing.

The scalability of the proposed algorithm, as demonstrated by its performance across different network sizes and configurations, demonstrates its potential for real-world deployment. By reducing computational complexity while ensuring effective resource allocation, the ADMM-based strategy can be implemented in large-scale satellite networks without incurring excessive computational overhead. This makes it a viable solution for next-generation satellite communication systems.

Although we have not explicitly graphed interference-related metrics (e.g., SINR, interference probability) in the outcome figures, the ADMM-based scheme inherently addresses co-channel interference by coordinating resource usage among multiple satellites and user terminals. The improved QoS metrics (notably throughput and latency) corroborate that our allocation decisions prevent severe interference conditions and maintain service quality. Specifically, by incorporating interference factors into our objective and constraints, the proposed algorithm actively confines interference levels to permissible ranges. Future research efforts could place additional emphasis on quantifying these effects through dedicated metrics, building on the foundational resource coordination framework presented here.

While our simulation results demonstrate the effectiveness of the proposed ADMM-based resource allocation and interference coordination strategy, conducting real-world experiments remains a challenge due to significant infrastructure, regulatory, and cost barriers. Future work will focus on exploring collaborations with industry partners to facilitate field trials, which would provide valuable insights into the practical implementation of our method and its performance in operational satellite networks.

## 6. Conclusions and Future Works

This paper has comprehensively addressed the joint dynamic task offloading and resource scheduling problem in LEO (LEO) satellite edge computing networks. Our findings underscore the importance of leveraging both LEO and GEO satellites in future network designs, as LEO satellites can provide both computational resources and efficient data transmission, enhancing overall network performance. The proposed system model incorporates both data service transmission and computational task offloading, framed as a long-term cost function minimization problem with constraints. Key contributions include the development of a priority-based policy adjustment algorithm to handle transmission scheduling conflicts and a DQN-based algorithm for dynamic task offloading and computation scheduling. These methods are integrated into a joint scheduling strategy that optimizes overall system performance. Simulation results demonstrate significant improvements in average system cost, queue length, energy consumption, and task completion rate compared to baseline strategies, highlighting the strategy’s effectiveness and efficiency. Future work will extend the framework to more complex network scenarios and explore the integration of machine learning with traditional optimization methods to further enhance performance.

In future research, we intend to investigate more advanced machine learning methods that can be combined with traditional optimization frameworks to further enhance resource allocation and interference management in heterogeneous satellite networks. For example, integrating deep reinforcement learning (DRL) techniques with our ADMM-based solution can help the network adapt to rapidly changing channel conditions and user demands by continuously learning optimal actions from environmental feedback. Similarly, multi-agent RL can be employed to coordinate decisions across multiple network entities (e.g., LEO, GEO satellites, and edge nodes), potentially accelerating convergence and improving overall system performance. Beyond RL, leveraging supervised or unsupervised learning methods for traffic prediction, node clustering, or link reliability assessment could also complement our optimization models, providing richer insights into network dynamics. These hybrid approaches—blending the reliability of mathematical optimization with the adaptability of machine learning—present promising avenues for future enhancement of resource management strategies in dual-layer satellite networks. 

## Figures and Tables

**Table 1:** Comparison of References in Section 2.

Ref.	Key Strength	Main Limitation
Part 2.1: Resource Allocation Strategies		
[10]	Improves energy efficiency and reduces traffic load	Lacks interference handling
[11]	Reduces latency with caching and MEC integration	GEO–LEO scalability not addressed
[12]	Enhances QoE with adaptive video streaming	Focused on terrestrial systems
[13]	Reduces delay using caching techniques	Limited satellite network considerations
[14]	Enhances multimedia resource allocation performance	Lacks multi-layer satellite focus
[15]	Balances resource scheduling between layers	Limited to static scheduling
[16]	Minimizes delay and offloading time using MEC	Assumes ideal network conditions
[17]	Improves spectral efficiency with NOMA integration	Limited to NOMA-based systems
[18]	Extends device battery life in IoT networks	Focused only on IoT traffic
[19]	Optimizes caching and computation offloading	Lacks satellite-specific integration
[20]	Simplifies hybrid SDN network management	Limited to flow table control
[21]	Integrates network, caching, and computing resources	Limited real-world validation
[22]	Dynamically improves QoE for real-time services	Focuses on terrestrial RAN
[23]	Balances energy use and QoE for video streaming	Targets only video traffic scenarios
Part 2.2: Interference Coordination Strategies		
[7]	Proposes effective spectrum-sharing approaches	Ignores dual-layer satellite interference
[8]	Reduces interference with sidelobe control	Limited to Ku-band
[9]	Provides ITU standards for spectrum sharing	Lacks dynamic technical solutions
[24]	Details uplink interference analysis in LEO-GEO networks	No resource optimization proposals
[25]	Analyzes downlink constraints in hybrid networks	Lacks layered control mechanisms
[26,27]	Offer regulatory interference coordination frameworks	Lack actionable algorithmic strategies
[28]	Suggests spatial isolation techniques	Ignores broader strategies
[29]	ITU-R global standards for spectrum sharing	No technical implementation methods
[30]	Simplifies RFI estimation in NGSO systems	Lacks mitigation techniques
[31]	Controls interference with joint power and tilt control	Requires complex coordination efforts
[32]	Improves coexistence with optimal power control	Limited to power-focused solutions
[33]	Enhances spectrum sharing with cooperative strategies	Lacks caching/computation integration
[34]	Maximizes spectral efficiency using NOMA pairing	Constrained to NOMA-specific systems

**Table 2:** GEO–LEO satellite interference thresholds [29].

Scenario	Frequency (GHz)	EPFD Threshold (dB(W/m^2^))
Uplink	28.6–29.1	−162
Downlink	18.8–19.3	−164

**Table 3:** Simulation setups.

Parameter	Value
GEO orbit height	35,786 km
LEO orbit height	1200 km
Earth radius	6371 km
Orbit inclination angle	87°
LEO satellite antenna gain	25 dBi
GEO satellite antenna gain	25 dBi
LEO ground station antenna gain	45 dBi
GEO ground station antenna gain	30 dBi

## Data Availability

The data that support the findings of this study are available upon reasonable request from the authors.
